# Ethnobotanical survey of medicinal wild plants in the Shouf Biosphere Reserve, Lebanon

**DOI:** 10.1186/s13002-022-00568-y

**Published:** 2022-12-26

**Authors:** Nizar Hani, Safaa Baydoun, Hatem Nasser, Tiziana Ulian, Nelly Arnold-Apostolides

**Affiliations:** 1 Faculty of Agriculture and Food Science, Holy Spirit University of Kaslik, Kaslik, Lebanon; 2 Research Center for Environment and Development, Beirut Arab University, Beirut, Lebanon; 3 Royal Botanic Gardens, Kew, Wakehurst, Ardingly, Haywards Heath, West Sussex, RH17 6TN UK

**Keywords:** Folk medicine, Traditional knowledge, Herbal remedies, Protected area, Mediterranean

## Abstract

**Background:**

Medicinal plants and associated traditional knowledge play a vital role in supporting the livelihoods and resilience of indigenous communities. This ethnobotanical survey aims to identify medicinal plants used by the local communities of the Shouf Biosphere Reserve of Lebanon (SBR) and document the associated traditional knowledge.

**Methodology:**

Focus groups and personal interviews with 133 informants of community members of 22 villages of SBR were performed during 2019–2022. Informants were selected using purposive sampling techniques based on their knowledge of medicinal plants and experience in traditional herbal medicine. Interviews were conducted using a semi-structured questionnaire through field visits.

**Results:**

Informants were equally represented by females and males and had different demographic characteristics, and the main source of knowledge was ancestral. A total of 184 medicinal plant species belonging to 57 families were documented. The predominant families were Asteraceae (31 spp.), Lamiaceae (14 spp.), and Rosaceae (14 spp.). Leaves (23%) were the plant part most used. Decoction (45%) was the predominant preparation method, while internal (oral) use (47%) was the most frequent administration mean. *Berberis libanotica, Dittrichia viscosa,* and *Daucus carota* achieved the highest scores of frequency of citation (FC), relative frequency of citation (RFC), use value (UV), and fidelity level (FL). Furthermore, diseases and ailments of gastrointestinal tract were the category most treated.

**Conclusions:**

Findings revealed a rich and diverse list of medicinal plants with associated traditional knowledge still actively used to treat a wide range of diseases. Future phytochemical and pharmacological studies are recommended to determine the efficacy and safety of plant species used. The management body of the SBR and all related authorities are invited to continue their conservation efforts to protect such rich biocultural heritage.

**Supplementary Information:**

The online version contains supplementary material available at 10.1186/s13002-022-00568-y.

## Introduction

Since antiquity, the use of medicinal wild plants has been globally practiced as one of the fundamental bases of traditional healthcare systems of indigenous communities [1]. Today, medicinal wild plants continue to provide a wide range of therapeutic benefits for many diseases and act as an important source of novel drugs and natural products [2]. The WHO Traditional Medicine Strategy 2014–2023 aims to strengthen the role of traditional medicine and emphasizes that traditional treatments, herbal medicines and traditional practitioners are a primary source, if not the only source, of health care for millions of people [3]. This intimate interaction between man and plants has led to the accumulation of a wealth of traditional knowledge presently recognized as relevant to preserving plant biodiversity and understanding the dynamic relationships between wild plants, social, and cultural systems [4]. Yet, this traditional knowledge is under the threat of erosion being mostly orally transmitted between generations and traditional practitioners [5]. Thus, documentation of this traditional knowledge through ethnobotanical surveys is important to preserve this valuable knowledge and valorize priority medicinal plants of high therapeutic potential toward new drug discovery and contribution to livelihoods of indigenous communities worldwide.

Situated in the Eastern Mediterranean, Lebanon represents a rich repository of medicinal plants and associated traditional knowledge [6]. The country hosts 360 species believed to have medicinal properties [7]. Many of these plants are extensively used in the preparation of herbal remedies that are used alone or in combination with conventional modern medications. These plants also serve as important raw materials for many natural products. Like many societies, it is becoming trendy in modern Lebanese societies to use combinations of traditional medicinal plants with conventional drug therapy [8]. This renewed interest may be classically seen in the context of changes in the lifestyle, that values the concept of natural products. Nevertheless, the observed upsurges in the prevalence of a wide range of both communicable diseases (CDs) and non-communicable diseases (NCDs) and epidemics among both Lebanese and Syrian refugees in recent years and current economic crises have encouraged many people to turn to medicinal plants. According to the World Life Expectancy data [9], cardiovascular diseases, specifically coronary heart disease, stroke, and hypertension, major diseases in Lebanon. While they account for 31% of worldwide deaths, these diseases cause 47% of all deaths in Lebanon with smoking being the major risk factor. With respect to CDs, the Lebanese Ministry of Public Health (LMOPH) epidemiological surveillance database has shown sharp increases in the prevalence of tuberculosis, measles, mumps, leishmaniasis, and hepatitis during the past 10 years [10]. Massive influx of Syrian refugees, combined with poor water management system, poor sanitation, deprived living conditions, and limited healthcare access in rural areas are reported as the main underlying causes of the upsurges. In addition to this heavy burden on the healthcare system in the country, the rapid spread of coronavirus disease 2019 (COVID-19), with several episodes and peaks [11] and current cholera outbreak now spreading all over the country have further exacerbated the pressure on the already fragile system [12]. Moreover, the recent STEPS survey of WHO in 2017 reports high prevalence of risk factors for NCDs in Lebanon [13]. The survey also reported that the financial and social burden of these diseases will grow dramatically in the next few years given the recent rise in population size. The inflation rate in Lebanon reached 162% during the last two years, dramatically affecting the healthcare systems in all districts in the country [14]. The country now faces a growing shortage of medical supplies and essential medicines (such as those used to treat chronic diseases), leaving the most vulnerable people at risk. With a current minimum wage of less than 30 USD, people have lost access to costly healthcare systems. Hospital admissions have also witnessed an unprecedented rise in patients presenting to emergency rooms because of clinical manifestations from their inability to afford diuretics, inhalers, antidiabetic drugs, and others [15]. In addition, social security institutions have only been able to cover 10% of the actual healthcare cost [16]. This diminished access of low- and medium-level income people to conventional medicines has encouraged many to turn to folk medicine and home care traditional medicine.

The above-mentioned circumstances have led many people of both rural and modern communities to perceive the use of medicinal plants as a healthy option for treatments [17]. In this context, previous ethnobotanical research on medicinal plants in some localities of Lebanon has documented some unique medicinal species such as *Salvia fruticosa* Mill.*, Achillea fragrantissima* (Forssk.) Sch.Bip.*, Artemisia herba-alba* Asso, and *Peganum harmala* L. These species are perceived to have a high potential therapeutic value in the treatment of many diseases such as diabetes, cardiovascular diseases, asthma, and rheumatism. Such studies provide the rationale for the selection and scientific investigation of priority species [18, 19, 20]. Nevertheless, research on this topic in Lebanon is very limited compared with other countries and still very restricted to specific areas. It is highly likely that many potential medicinal plants are still not explored yet and are waiting to be identified. Therefore, more research is needed to explore this potential in other regions in the country especially in forests given their richness of flora biodiversity and proven profitability of sustainable exploitation of medicinal plants [21].

In this respect, the forest of Shouf Biosphere Reserve (SBR) stands as a potentially rich resource of wild plants being one of the valuable ecosystem services of the Reserve. Estimated in the range of 16.7 to 21.3 million US dollars/year, SBR provides a variety of ecosystem services [22] including the direct use of plants of which medicinal plants continue to play a major role. Vegetation of SBR has a high diversity due to the altitudinal gradient, continentality gradient, lithological variability, and the historical interactions between people and nature [23]. Supra-Mediterranean (1000–1500 m.a.s.l.) and Oro-Mediterranean (1500–2000 m.a.s.l.) bio-climatic zones are represented in SBR and each zone corresponds to a series of plant communities. While the higher parts of SBR are rich in species that accompany the *Cedrus libani* A.Rich. forests such as *Prunus prostrata* Labill. and *Berberis libanotica* Ehrenb. ex C.K. Schneid. and the lower parts, where the communities live, are very rich in the medicinal species that accompany the agricultural lands such as *Ruscus aculeatus* L.*, Salvia sclarea* L. and *Hypericum triquetrifolium* Turra. The recent list of plant species in SBR identified 1054 different species.

**Fig. 1:**
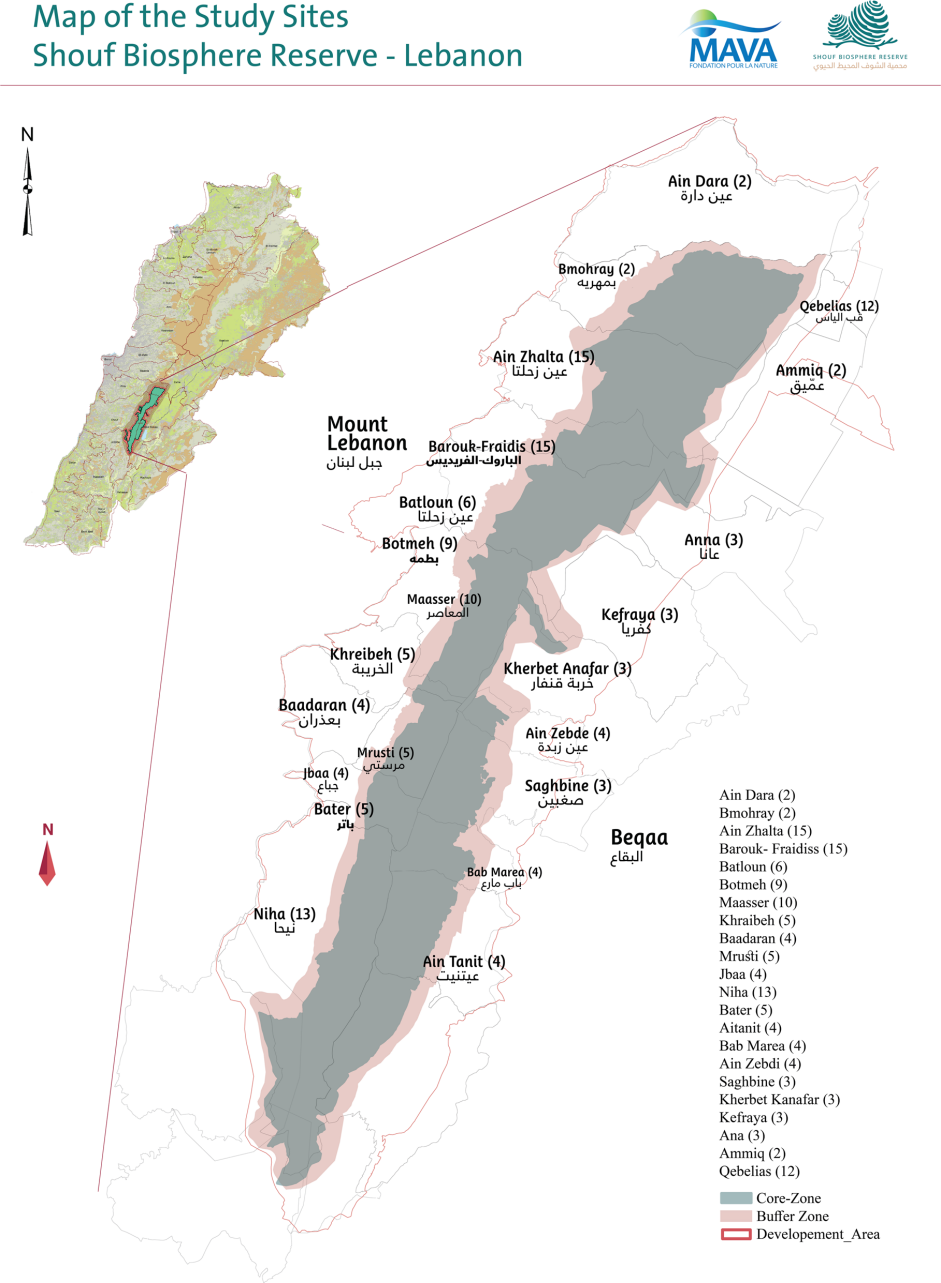
Study sites and distribution of informants: Ain Dara (2), Bmohray (2), Ain Zhalta (15), Barouk- Fraidiss (15), Batloun (6), Botmeh (9), Maasser (10), Khraibeh (5), Baadaran (4), Mrusti (5), Jbaa (4), Niha (13), Bater (5), Aitanit (4), Bab Marea (4), Ain Zebdi (4), Saghbine (3), Kherbet Kanafar (3), Kefraya (3), Ana (3), Ammiq (2), and Qebelias (12)

The SBR includes three zones (core, buffer, and development) (Fig. 1) and is home to more than 170,057 inhabitants, living in the 22 villages of the development zone. Security issues, political instability, Syria crisis, COVID-19, and the current economic situation have had a marked impact on the social fabric of SBR that hosts now approximately 58,000 Syrian refugees, mostly in its eastern side [23].

The healthcare system of communities of SBR includes primary care centers, dispensaries, private clinics, and a few hospitals in addition to the herbalists and home care traditional treatment, in which medicinal plants present a major contributor. The current economic crises compounded with the big number of refugees residing in the area have added extra pressure on the healthcare system. Such circumstances have encouraged local communities to revive their ethnobotanical medical system. It is observed that herbalists and local healers now receive more locals for traditional consultations and treatments.

In this direction, SBR has recently established the commercial production of the hydrolates and essential oils of several important medicinal plants such as *Eryngium creticum* Lam, *Origanum syriacum* L.*, S. fruticosa, Dittrichia viscosa* (L.) Greuter*, Urtica dioica* L., and *U. urens* L*.* Also, a Zaatar House (Oregano House) was established at one of Reserve's villages, (Maasser el Shouf), where the waters and essential oils of *O. syriacum* and *Lavandula angustifolia* Mill. are commercially produced and sold. Despite these important initiatives, there is still a need to have a good understanding of the medicinal plant species and associated traditional knowledge of SBR.

This ethnobotanical survey aims to identify medicinal plants used by the local communities of SBR and document related traditional knowledge. The following questions are addressed: What are the plant species local communities of SBR use in their traditional healthcare system? What are the associated practices? What are the disease categories treated by the identified plants? Findings could contribute to the scientific understanding of traditional medicine and identification of priority species of high potential for drug discovery, economic valorization, and livelihood development of local communities.

## Materials and methods

### Study area

The study area of this ethnobotanical survey is SBR (Fig. 1). SBR lies between longitude 35° 28′–35° 47′ east and latitude 33° 32′–35° 48′ north at an altitude of 800–1950 m. a.s.l. occupying an area of approximately 539 km^2^ equivalent to 5% of the total area of Lebanon, making it the largest protected area in the Mediterranean area of the Middle East. Designated by UNESCO in 2005, SBR consists of the Al-Shouf Cedar Nature Reserve that was established by the Lebanese Government in 1996 under the authority of the Ministry of Environment and 22 surrounding villages (i.e. Ain Dara, Bmohray, Ain Zhalta, Barouk-Fraidiss, Batloun, Botmeh, Maasser, Khraibeh, Baadaran, Mrusti, Jbaa, Niha, Bater, Aitanit, Bab Marea, Ain Zebdi, Saghbine, Kherbet Kanafar, Kefraya, Ana, Ammiq, and Qebelias) located in the Shouf, Aley, and West Bekaa districts [24]. The SBR is home to more than 170,057 inhabitants, spreading over villages, at 800–1200 m altitude a.s.l., located in the development zone of the Reserve that surrounds the core and buffer areas. The largest population (more than 50,000 inhabitants) lives in Qebelias town, while the smallest community lives in Bab Marea (less than 1000 inhabitants), and the average number of inhabitants in the study area villages is 3000–4000 inhabitants. Around 50–55% of the population lives all year round in the study area, the remaining comes during weekend and summer holidays, some of them live in Beirut, The capital city of Lebanon, and bigger cities, and the others are living outside Lebanon. On average 30% of the population are civil servants, 30% are engaged in agriculture activities, 25% are engaged in private businesses, and 15% are unemployed. The SBR is a multicultural region with a mosaic of religious communities and is the home of the Druze, settled since the Middle Ages, and to numerous Maronite Christians, Greek Catholics and Sunni Muslims [23]. Rich in history, having been the center of the Emirate of Mount Lebanon, the area hosts a wide choice of heritage sites of archeological places, historical palaces, and religious sites, all adding to the charm and attractiveness of this unique place.

Climate of SBR is characterized by a bio-climatic gradient from the supra-Mediterranean type at the lower altitudes, with fresh to cold temperatures and subhumid conditions (annual rainfall between 600 and 1300 mm), to the Oro-Mediterranean type at higher altitudes, with subhumid to humid conditions (annual rainfall between 1000 and 1600 mm) and cold to very cold temperatures. The eastern slopes have drier conditions with annual rainfall lower than 600 mm [25].

### Ethnobotanical survey and data collection

The data collection was performed during 2019–2021 following the different flowering seasons in the 22 villages of SBR by focus groups and personal interviews organized with key informants of community members. Random number of informants (1–15) was selected from each village by purposive sampling method based on the knowledge of informants about medicinal plants and experience in traditional herbal medicine. A semi-structured questionnaire was used in the interviews covering informant personal information (age, sex, education, and profession) and source of knowledge, vernacular name of plant species, ailments and diseases treated, and preparation and administration methods.

Data collection followed the code of ethics of the International Society of Ethnobotany [26] and was respected by the Convention of Biological Diversity [4] and the CBD Nagoya protocol on Access and Benefit Sharing [27]. Prior to the implementation of the study, the approval of SBR committee in terms of objectives, design, and approach and a verbal consent of each informant post providing a clear explanation of the study's objectives were obtained.

The interviews with the informants were mainly conducted during repetitive field walks mainly during the flowering season to ensure capturing the seasonal differences and the whole floral cycle. Alternatively, fresh specimens and photographs of plant species were shown to informants in case of limiting conditions. Photographs of cited species were taken, and some interviews were recorded using a mobile phone or filmed using a professional videographer. Voucher fresh floristic specimens were collected, pressed, and labeled immediately in the field and deposited at the SBR herbarium. These specimens were later scanned and saved online in a special database on the SBR Web site [28, 29].

Plant identification was conducted based on the taxonomic keys of the New Flora of Lebanon and Syria [30] and confirmed by the Botany, Pharmacognosy, and Pharmacology Professor Dr. Nelly Arnold Apostolides. New species naming and botanical family delimitation were based on Plants of the World Online [31]. Species ID was based on the nomenclature of the International Plant Names Index [32]. The Raunkiar's life form classification system was used to classify the plants in this survey into herbs, climbers, shrubs, and trees [33].

### Ailment and disease categories

All ailments and diseases cited by informants were classified into categories according to the International Classification of Primary Care (ICPC-2) (https://www.icpc-3.info/), post some modifications (Table 4). Indications, such as headache, toothache, poisoning (as antidote), scurvy, cancer, eye infections, allergy, and fatigue, that did not match with any of the categories were grouped in the "Others category".

### Data analysis

Quantitative analysis of collected data was performed based on computing the following indices:

- *Frequency of Citation (FC)*
  Number of use reports (time a particular species was mentioned)/total number of times that all species are cited × 100.
- *Relative frequency of citation (RFC)*
  Number of informants mentioning the use of a species divided by total number of informants participating in the survey [34]. The RFC index ranges from "0" when no citation of a plant species as useful to "1" when all informants refer to the plant as useful.
- *Use value (UV)*
  This index quantifies the relative importance of cited plants [35, 36]. It is calculated by the formula UV = ∑ U/N, where "U" refers to the number of uses mentioned by the informants for a species and "N" refers to the total number of informants interviewed. A high UV score indicates there are many use reports for that plant, while a low score indicates fewer use reports cited by the informants.
- *Factor of informant consensus (FIC)*
  This index reflects the level of homogeneity between information provided by different informants. It was calculated as FIC = Nur − Nt/(Nur − 1) [37], where Nur is the number of use reports from informants for a particular disease category and Nt is the total number of species used for that category.
- *Fidelity level (FL)*
  This index is the percentage of informants who mention the use(s) of a certain plant species to treat a particular ailment or disease. It quantifies the informants' choice for a potential plant species to treat a given ailment, thus reflecting its therapeutic importance (ref). It is calculated using the formula: FL (%) = Np/N × 100 , where Np is the number of informants that claimed use of a particular plant species for a particular ailment or disease and N is the total number of informants citing the species for any ailment or disease. The maximum FL illustrates the frequency and high use of the species for treating a particular ailment by the informants in the area under study.

## Results

### Demographic features of informants and source of knowledge

A total of 133 informants of different demographic groups were interviewed on the therapeutic properties of medicinal plants throughout the 22 villages under study (Table 1). As indicated in the table, informants were almost of equal shares of women and men. They constituted five age groups falling in the range of 19–97 years with the majority being 40–80 years. Informants were also of different educational backgrounds. Farmers and housewives together represent the majority of informants' professions, whereas other professions were exemplified by a few persons only.

**Table 1:** Informants' demographic data, source of knowledge, and distribution of ethnobotanical data (number and percentage of cited species)

Demographic features	Number (%)	Botanical data %	Demographic features	Number (%)	Botanical data %
**Gender**			**Educational level**		
Men	65 (49%)	58	University	25 (19%)	10
Women	68 (51%)	42	Secondary	40 (30%)	21
**Source of knowledge**			Intermediate	52 (39%)	43
Ancestral	94 (71%)		Primary	4 (3%)	18
Self-Training	34 (25%)		No schooling	12 (9%)	10
Traditional practitioners (TP)	5 (4%)				
**Age group**			**Profession**		
< 20 years of age	1 (1%)	0.5	Farmers	60 (44%)	44
20–39 years of age	4 (3%)	1	Shepherds	3 (2%)	1
40–59 years of age	55 (41%)	39	Housewives	54 (41%)	38
60–79 years of age	58 (44%)	52	Shopkeepers	5 (4%)	2
> 80 years of age	15 (11%)	7	Traditional practitioners (TP)	5 (4%)	13
**Religious background**			Healthcare professionals (dentist, pharmacist, nurse)	7 (5%)	2
Muslim Druze	107 (80%)	81			
Muslim Sunni	16 (12%)	13			
Christians	10 (8%)	5			

Table 1 also shows the percentage of botanical data per informant group. While males provided 58% of the cited species, females presented the remaining number (42%). Older age groups cited more plants than younger age groups with most plants (89%) being cited by informants of the 40–80 years. Moreover, 71% of cited plants were provided by informants who had lower level of education (i.e., no schooling, primary, and intermediate levels). As for the profession aspect, farmers and housewives representing 85% of the total number of informants, provided 82% of the cited plants. Despite their small number (5 informants, 4%), traditional practitioners provided 13% of the cited plants.

The pattern of distribution of informants’ knowledge related to religious background shows that the majority of species (81%) were cited by Druze informants. Whereas, each of Sunni and Christians provided 13% and 6%, respectively, noting that all the cited species by the latter groups were common with the former one. Among these species were *Salvia fruticose* for colic and stomach/intestinal troubles, *Matricaria aurea* (Loefl.) Sch.Bip., *Micromeria myrtifolia*, and *Myrtus communis*, for cold and flu. Interestingly, some informants of the Druze religious group identified a few species such as *Acantholimon libanoticum* Boiss. and *Laurus nobilis* L. for their importance in spiritual protection against diseases and witchcraft. As for the main source of informant's knowledge, oral transmission of ancestors (71%) was reported as the main approach through which informants acquired their knowledge about the medicinal properties of plants and means to use them. But, self-training using traditional manuscripts, modern online means, and contemporary books represented 25% and learning from fellow herbalists and traditional practitioners formed 4% only.

### Cited species diversity

The floristic analysis of cited species reveals a list of 184 plant species of medicinal properties used for the treatment of a wide range of diseases. Additional file 1: Table S1 (quantitative and qualitative ethnobotanical data of wild medicinal plants in the Shouf Biosphere Reserve, Lebanon) presents the scientific and common names, botanical families, and life forms of these species. It also presents plant parts used, therapeutic indications, preparations and administrations, dosages, along with quantitative analysis values (FC, RFC, UV, RI, and FL) used as a tool for the prioritization of certain species for future pharmacological research and development projects. Remarks provided by informants such as other uses, toxicity, and endemism are also included.

The cited species belonged to 57 families with Asteraceae (31 spp.) having the predominant representation, followed by Lamiaceae (14 spp.), Rosaceae (14 spp.), Fabaceae (7 spp.), Apiaceae (7 spp.), Caryophyllaceae (6 spp.), and Brassicaceae (6 spp.). Other families were represented by no more than four species only.

Based on the New Flora of Lebanon and Syria [30], all the 184 cited species were native. Among them, two species, i.e., *Berberis libanotica* and *Pseudopodospermum libanoticum* (Boiss.), were endemic to Lebanon–Syria, and another two, i.e., *Arum palaestinum* Boiss. and *Onopordum cynarocephalum* Boiss. & C.I. Blanche were endemic to Lebanon–Syria–Palestine. Also, 15 other species were endemic to the region including Turkey, Cyprus, Greece and Iraq, i.e., *Acanthus hirsutus* subsp. *Syriacus* (Boiss.) Brummitt*, Achillea falcata* L.*, Crocus graveolens* Boiss. & Reut.*, Ferulago trachycarpa* Boiss. and *Valeriana erotica* Christenh. & Byng.

Herbaceous species constituted the highest contribution (156 species) of the cited species, while climbers, shrubs, and trees contributed 2, 11, and 15 species, respectively.

### Mode of preparation, administration methods, and dosage of application

Most plant species were cited for the treatment of more than one ailment and disease, for instance, *Matricaria aurea* (Loefl.) Sch.Bip treated 24 conditions, *Taraxacum officinale* Weber, 23 conditions and *Urtica urens* L. 19 conditions (Additional file 1: Table S1).

Leaves represented the plant parts most used (23%), followed by aerial parts (15%), flowers/inflorescences (14%), roots/bulbs/rhizome/tubers (14%), and fruits (10%) (Table 2). Other parts such as whole plant, seeds, bark, wood, latex, and stem were also cited. For most plants, more than one plant part was used.

**Table 2:** Number and percentage of cited species of different plant parts used

Plant parts	Whole plant	Aerial parts	Flowers/inflorescences	Leaves	Fruits	Seeds	Bark	Resin/gum	Roots/bulb/rhizome/tubers	Wood	Latex	Stem/young shoot
No. species	36	61	56	91	39	28	8	6	58	3	3	15
%	9	15	14	23	10	7	2	1	14	1	1	4

Preparation methods were diverse with decoction being most common (45%), followed by infusion (25%), maceration (9%), and latex/fresh juice (9%) (Table 3). Informants also reported the use of excipients (water, alcohol, oil, honey, and eggs) and some additives (salt and sugar).

**Table 3:** Number and percentage of species of different preparation modes

Preparation mode	Decoction	Infusion	Maceration	Latex/fresh juice/syrup	Hydro-distillation	Fumigation	Steaming	Powder
No. species	123	67	25	25	11	5	4	12
%	45	25	9	9	4	2	1	4

While most preparations were based on the use of a single species, some mixtures for treating respiratory and gastrointestinal conditions, in particular, were also commonly reported. For example, a mixture of *Micromeria myrtifolia* Boiss. & Hoben.*, Myrtus communis* subsp. communis, *Matricaria aurea, Salvia fruticosa*, *Alcea setosa* (Boiss.) Alef., and *Mentha longifolia (*L.) L. was used to treat common cold, cough, flu, and sore throat. Another common mixture was made of *Salvia fruticosa*, *Origanum syriacum*, and *Foeniculum vulgare* Mill. This mixture was used for stomach ache and colic conditions. In the case of diarrhea, some peels of pomegranate (*Punica granatum* L.) were added.

The majority of remedies were prepared from fresh materials soonest after collection (60%), and some were prepared from either dried or fresh materials (31%), while a few were only used from dried materials (9%).

Medicinal formulations were administered orally (internally) (47%) in ailment categories other than dermatological problems, 8% was administered externally in dermatological ailments, while both usages internally and externally were used in 45% of cited plants.

Informants used different measuring units such as Arabic coffee cup (70 ml), glass (200 ml), soup bowl (350 ml), teaspoon (6 ml) or tablespoon (12 ml), pinch of three fingers (5 g), and handful (20 g), in addition to traditional and contemporary weighing measures such as oz (28 g) and dirham (3 g). Formulations were taken till recovery.

Interestingly, dosage of detailed formulations was particularly provided by some traditional portioners. For example, one coffee cup per day for 15 days of the decoction of *Daucus carota* seeds was recommended for cystitis, diuretic, laxative, diabetes, spasms, etc. (Additional file 1: Table S1). Also, a dose of two cups per day (morning and evening) for two days only of the decoction of the seeds mixed with cherry peduncles and palm dates was cited as highly efficient in the treatment of kidney stones (Additional file 1: Table S1).

Nevertheless, informants expressed concern about side effects of some species in cases of overuse (Additional file 1: Table S1). Among the most toxic species were such as *Arum palaestinum* Boiss*., Arum hygrophilum* Boiss*., Eupatorium cannabinum* L*., Ruscus aculeatus* L.*, Dittrichia graveolens* (L.) Greuter*, Ricinus communis* L*., Tussilago farfara* L.*, Lonicera etrusca* San.*, Ecballium elaterium* (L.) A.Rich*, Hyoscyamus aureus* L.*,* and *Prunus cocomilia* Ten*.* In particular, ingestion of a high dose of *E. cannabinum,* a species *cited* for the treatment of liver and gallbladder disorders, skin infections, colds, and fever causes tremor, delirium, and even death. Also, a relatively high frequent smoking of *T. farfara* leaves was perceived as a seriously harmful practice. Likewise, *E. elaterium* was considered highly toxic if a dose greater than one drop/day is administered as a nasal instillation. Such a dose causes serious side effects such as uvular edema, nasal and tongue edema, and other adverse effects on the voice of users. Also, a high dose of *P. cocomilia* seeds and young shoots used for diarrhea, skin diseases, liver cirrhosis, and diuretic was cited as toxic. With this particular example, informants reported the use of the level of bitterness as an indicator of toxicity, while berries of the *L. etrusca* were cited among the mildly poisonous.

Further, informants highlighted the multiuse of several cited species (Additional file 1: Table S1). The diverse uses reported were: edible for human, ritual and spiritual use, ornamental and decoration, source of natural materials (cosmetics, insect repellent and pesticide, dyes), firewood, forage, and melliferous. *Gundelia tournefortii* L.*, Pseudopodospermum molle* (M.Bieb.) Kuth.*, Eryngium creticum, Rhus coriaria* L.*,* and *Cichorium intybus* L. among others (*n* = 84 species) were cited as edible plants. Further, *Anchusa hybrida* Ten*., Calicotome villosa* (Poir.) Link and *Cedrus libani* were among the most frequently cited as melliferous plants (*n* = 33). Also*, Cistus creticus* L. and *Chrozophora tinctoria* (L.) A. Juss. were among species cited for forage. Informants also reported that treatment was done by oneself in common ailments such as common cold, indigestion, mosquito bite, and abdominal pain.

### Ethnobotanical indices

**Fig. 2:**
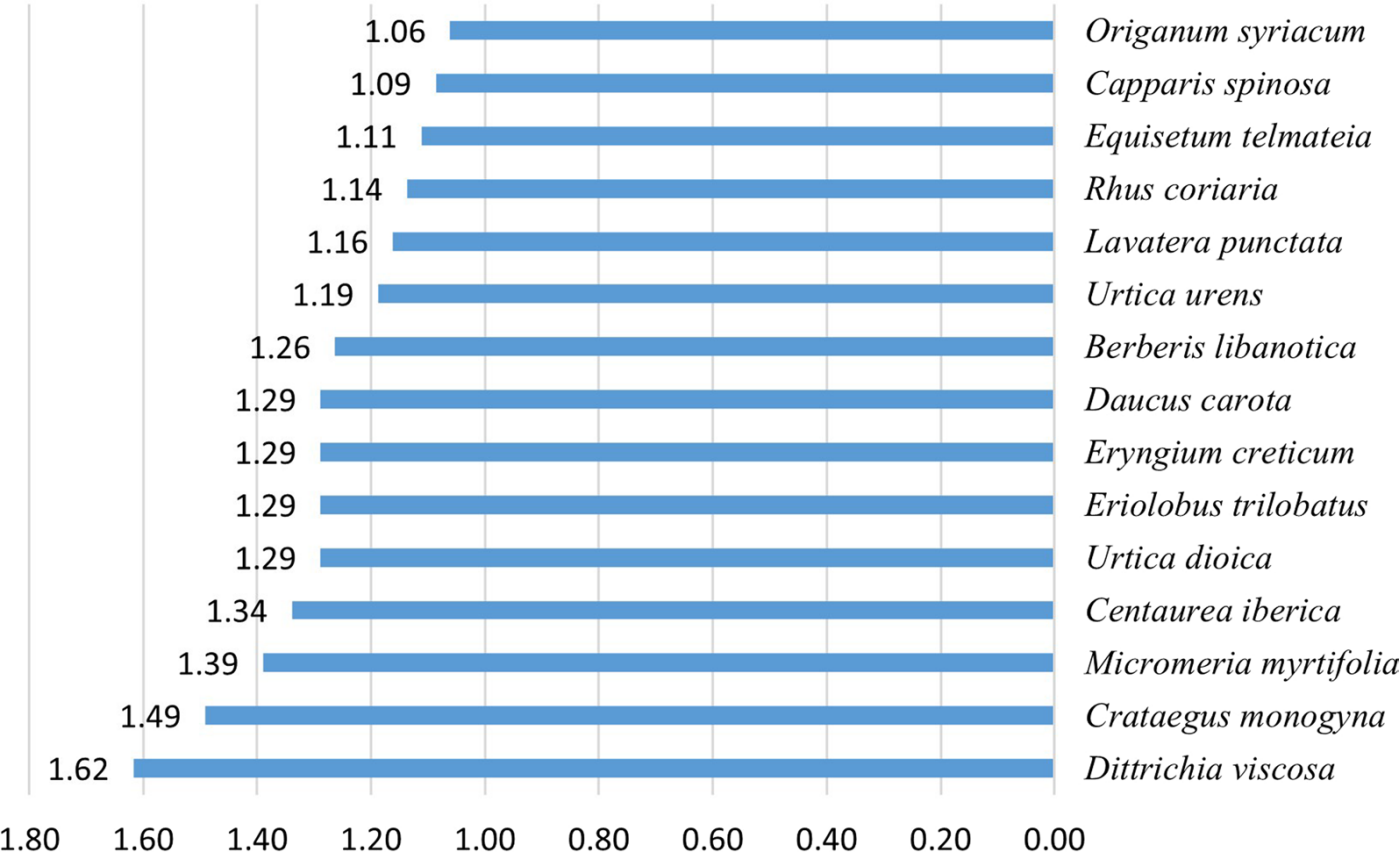
FC values for the top 15 species

Quantitative indices presented in Additional file 1: Table S1 and Fig. 2 revealed that species of highest FC values were *Berberis libanotica, Hyoscyamus reticulatus, Dittrichia viscosa, Echinops spinosissimus*, and *Urtica dioica.*

**Fig. 3:**
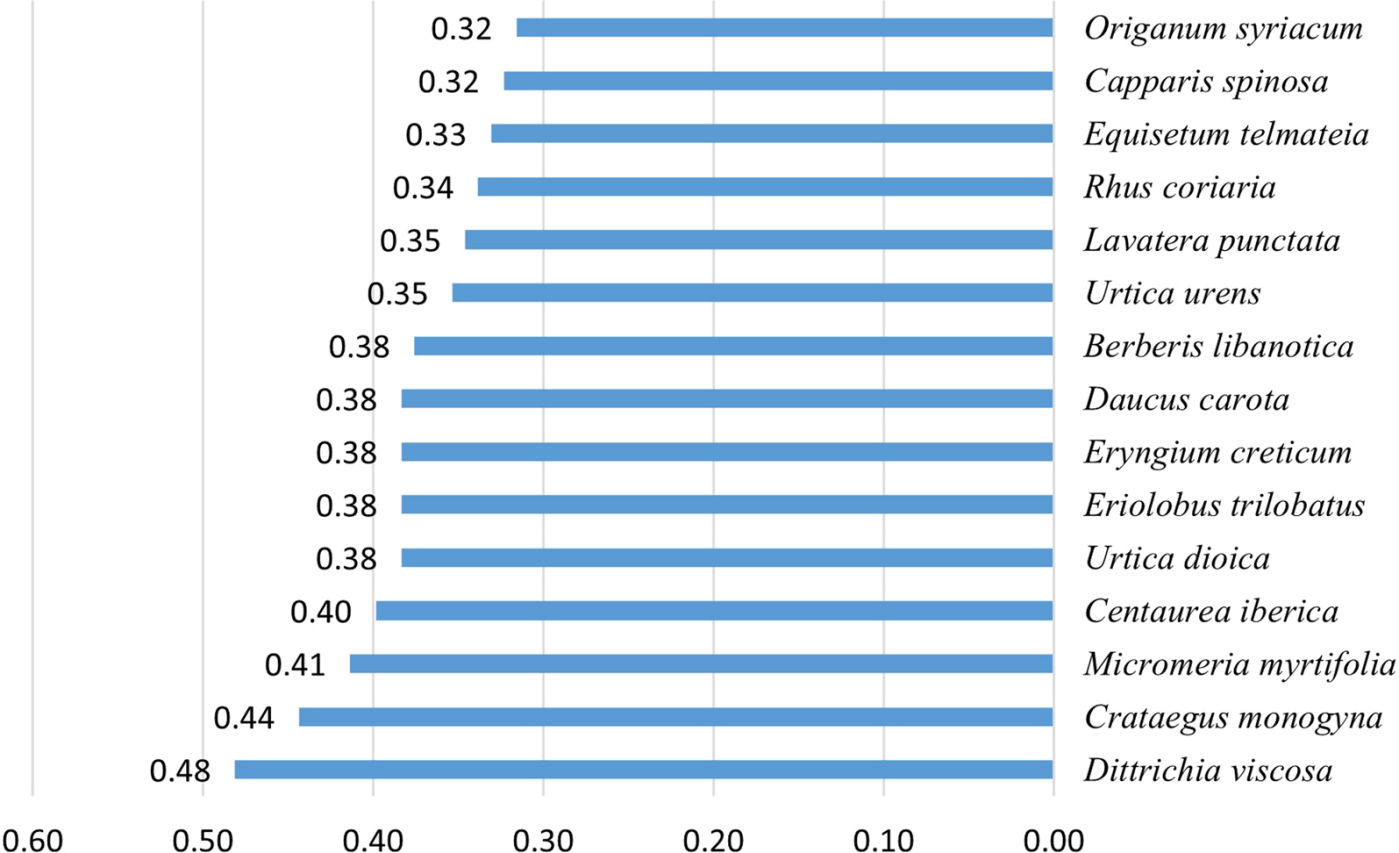
FCR values for the top 15 species

Additional file 1: Table S1 and Fig. 3 also show that RFC ranged between 0.05 and 0.48 with *Dittrichia viscosa* scoring the top value. Other high RFC values were achieved by *Capparis spinosa* (0.44)*, Micromeria myrtifolia* (0.41), *Centaurea iberica* Trevir. ex Spreng. (0.40), and *Daucus carota* L. (0.38). Species of low RFC included *Blitum virgatum* L. and *Aristolochia sempervirens* L. having the value of 0.05.

**Fig. 4:**
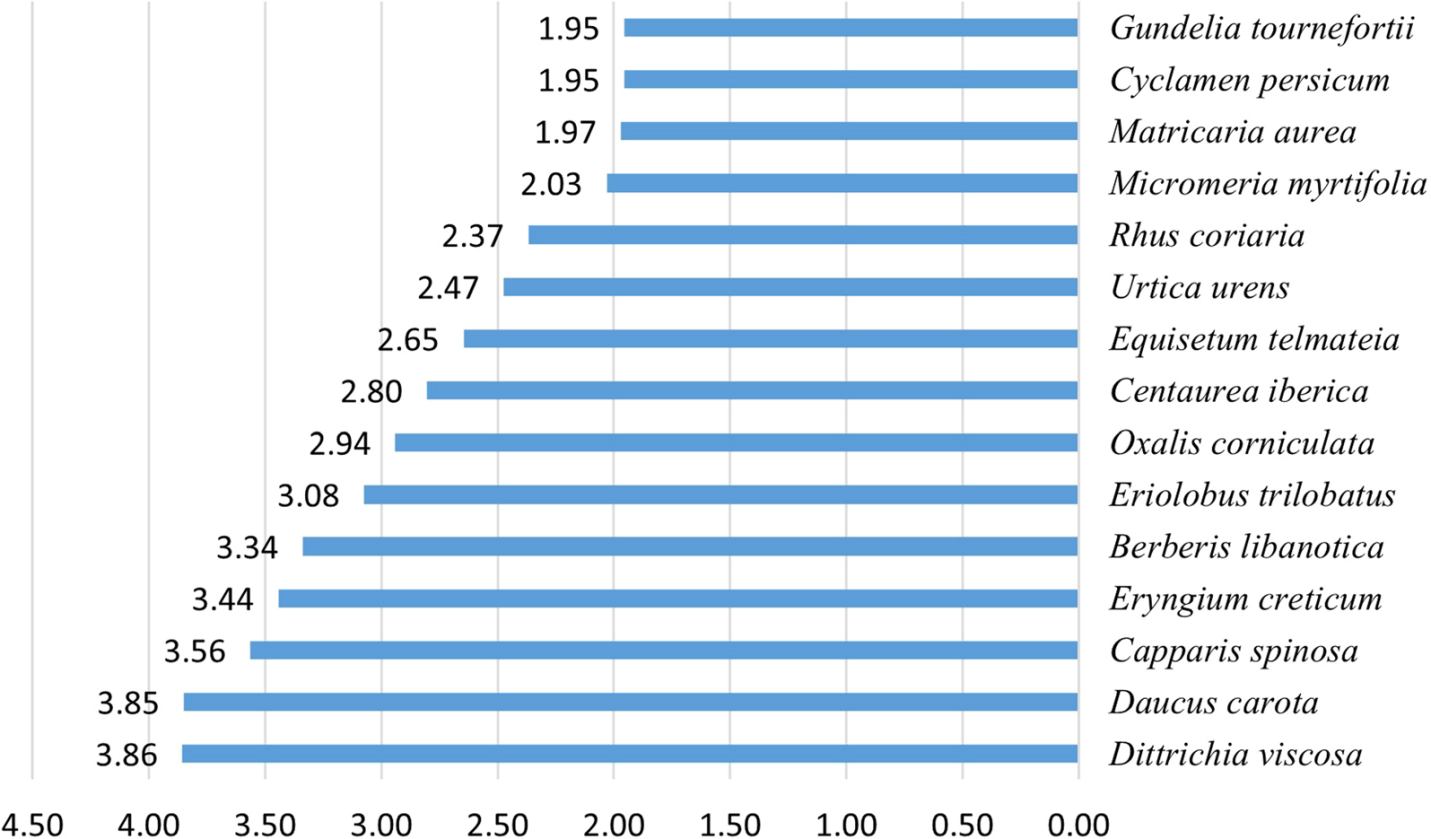
UV values for the top 15 species

Scores of UVs ranged between 0.10 and 3.86 with *Dittrichia viscosa* achieving the highest score (UV 3.86). The plant was cited to treat a wide range of diseases and ailments including skin diseases (wounds and infections), respiratory (bronchitis), gastrointestinal and liver (stomach ulcers), diabetes, and rheumatism. *Daucus carota* followed with a score of UV 3.85 cited for the treatment of a less number of ailments (Additional file 1: Table S1 and Fig. 4). On the other hand, *Valeriana rubra* L*.* and *Ranunculus ficarioides* Bory & Chaub. achieved the lowest values (UV 0.10).

Wide diversity of diseases and ailments was recorded and grouped into 14 use categories (Table 4). Gastrointestinal system and liver diseases were treated by 16% of the cited plants. This was followed by skin and related symptoms (13%), respiratory system diseases (9%), central nervous system (7%), and blood–hematopoietic system (7%) (Table 4). Accordingly, the FIC values reflecting the agreement between informants on the use of a species in the treatment of specific disease categories fell in the 0.62–0.02 range.

**Table 4:** The categories of diseases and ailments treated by medicinal plants of SBR and relevant FIC values

Category	Number of plants	FIC
1. Respiratory system: Cold, sinusitis, sore throat, cough and asthma, catarrhs, etc	83	0.58
2. Gastrointestinal system and liver disease: Diarrhea, intestinal worms, abdominal cramps, gastric ulcers, aerocoly, hemorrhoids, etc.	142	0.62
3. Renal system: Kidney stones, renal infections, renal insufficiency, edema, etc.	34	0.39
4. Genital system: Impotency and sterility, menstrual disorders, prostate disorders, uterine infections, etc.	50	0.26
5. Fever	28	0.1
6. Blood–hematopoietic system: Gout, blood neoplasms, anemia, hypercholesterolemia,	64	0.33
7. Diabetes	48	0.02
8. Skin and related symptoms: Ecchymosis, wounds, skin diseases, vitiligo, warts, insect stings, headlice, etc.	118	0.56
9. Rheumatism	37	0.05
10. Central nervous system: Insomnia, epilepsy, sciatica, etc.	64	0.43
11. Infectious and urinary diseases: Infections	74	0.26
12. Cardiovascular system: Hypertension, palpitation	24	0.26
13. Inflammation	50	0.21
14. Others: Headache, toothache, poisoning (as antidote), scurvy, cancer, eye infections, allergy, and fatigue	91	0.36

**Fig. 5:**
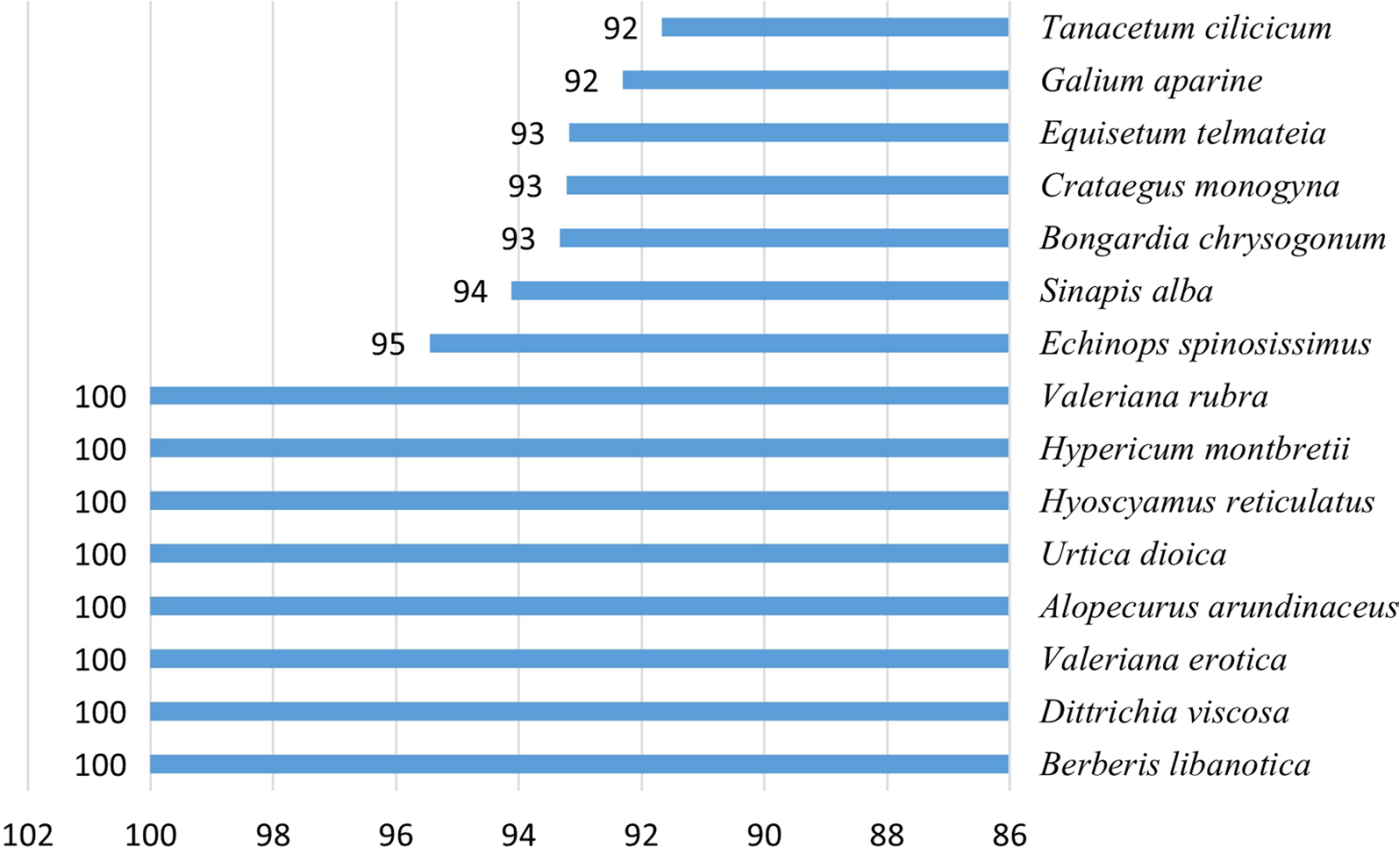
FL values for the top 15 species

The highest fidelity level (100%) was recorded with *Berberis libanotica, Hyoscyamus reticulatus* L., and *Dittrichia viscosa* which were frequently cited to treat specific ailments and diseases including respiratory, skin, gastrointestinal, blood–hematopoietic system, diabetes, and rheumatism and ranged between 90 and 95% for the rest of highly cited plants (Additional file 1: Table S1) (Fig. 5).

## Discussion

In this study, a rich list of 184 medicinal plant species belonging to 57 botanical families was reported by 133 informants from the local communities of the SBR for use in the treatment of 14 categories of ailments and diseases. The rich list of medicinal plants highlights the importance of the provision of ecosystem services of SBR that support healthcare systems and the well-being of communities in the 22 villages of the SBR. This high number of cited species and associated traditional knowledge clearly reflects that medicinal plants continue to play a critical role in the healthcare systems of these communities and present a potential resource for resilience and sustainability. This is especially relevant in the times of the economic crises Lebanon is presently facing and the growing need for national production for health and pharmaceutical industries in the country.

### Demographic feature of informants and source of knowledge

The results presented in Table 1 suggest that the demographic variables of informants influence the traditional botanical knowledge of cited medicinal plants. Despite their nearly equal numbers in the inquiry under study, men seem to have more knowledge as they provided 58% of the collected data compared with women. This pattern may be related to the broader exposure men have while practicing their professions and communicating with community members and fellows of the same professions. This may be indicated in the fact that a significant share of reported knowledge was provided by farmers being all males who can acquire good botanical knowledge about the healing effects of plants through their observations of the animals' responses to plants consumption and detection of the side effects and properties of different plant species to control several pests that attack crops. Nevertheless, housewives also provided a high share of knowledge (38%) that can be attributed to their role in taking care of their families and their high tendency to develop a cohesive social network with other women and share knowledge with their ancestors. Data on age groups clearly show that older people had much experience, skills, and knowledge on therapeutic properties, preparation, and administration of remedies related to cited plants. The drop of knowledge noted among younger generations may be due to changing lifestyles and transitions in traditional medicine interests. Data also show that education seems to have an inverse relationship with knowledge as many educated people do not have an interest to learn and practice traditional use of medicinal plants probably because time invested in schooling deflects them from acquiring botanical knowledge of medicinal plants. The aforementioned influence of demographic characteristics of local communities on botanical knowledge is commonly reported in the literature [38]. Actually, the acquisition of traditional knowledge on medicinal plants is believed to be a complex and dynamic social process that can be influenced by multiple socioeconomic and cultural drivers that mostly do not act independently [39]. Nevertheless, the dominance of Druse in the distribution pattern of ethnobotanical knowledge related to religious groups comes as no surprise since the majority of informants consisted of this group. However, this dominance may disguise certain variations related to levels of reliance on natural resources, types of cultural bonds to the land, and other factors between different religious groups. Given the fact that ethnobotanical knowledge is fast disappearing, it therefore becomes important that the influence of socioeconomic variables including religions on ethnobotanical knowledge of medicinal plants is urgently well examined and recorded [40].

Further, the dominance of oral transmission of ancestors as the main sources of ethnobotanical knowledge (65%) may be associated with the fact that communities in SBR villages are characterized by deep traditional customs and that information about herbal medicines is often considered secret, and therefore, only transmitted to descendant by elders. This indicates a high risk of loss of such important cultural heritage, and the need is to devise reliable means to avoid this risk.

### Ethnobotanical diversity

The high diversity of plants identified in terms of total number and representative families may be mostly due to the high diversity and abundance of these species and their botanical families in Lebanon and neighboring countries in the Eastern Mediterranean (Additional file 1: Table S1) [41–44]. Also, the effectiveness of members of these families may be of a high relevance. The literature shows that many members of these families have antioxidant, anti-inflammatory, antimicrobial, anti-hyperlipidemia, vasorelaxant, antithrombotic, hepatoprotective, and diuretic effects. These bioactivities are attributed to the phytochemical components of relevant species [45–47]. For example, the chemical composition of *Matricaria aurea* of Asteraceae is dominated by terpenoids and phenolic compounds, phenolic acid, flavonoids, and coumarins [48]. Another example of Asteraceae is *Dittrichia viscosa* which is rich in sesquiterpenes, flavonoids, and caffeic acids among others [49]. Also, the high diuretic activity of *Taraxacum sect. Taraxacum* F.H.Wigg*.* may be attributed to its high potassium content [50]. The astringent properties of tannins being a major constituent in Rosaceae species promote rapid healing and the formation of new tissues on wounds and inflamed mucosa [51]. Tannins are effective in the treatment of diarrhea, intestinal catarrh, varicose ulcers, hemorrhoids, minor burns, frostbite, as well as inflammation of gums and as an antidote. Moreover, tannins are recently found to demonstrate antiviral activities for treatment of viral diseases including AIDS [52]. However, species of lower FC and RFC, such as *Blitum virgatum* L., *Smilax aspera* L., *Vitex agnus-castus* L., may also contain in their phytochemical profile a range of constituents that may potentially have some important therapeutic properties [53, 54, 55]. This calls for future comprehensive research to further explore the potential of such species as a source of novel drugs.

The most widely used species were of herbaceous life form constituting the highest category of 156 species (85%). This may be attributed to their relatively higher abundance in Mediterranean forest ecosystems being subjected to intense human transformation [30]. Also, collection of herbaceous plant material may be relatively less time and effort consuming. Similar findings were reported by other studies throughout the world [56, 57].

Most species were used in monotherapies based on preparations from a single plant to treat more than one condition. This may be attributed to the presence of several active components in the composition of one particular plant and also the fact that such components can be active against several pathogens. In the other occasions where mixtures of two or more species were used as efficient treatment of certain conditions indicate a potential synergistic effect of such plants.

Some of the medicinal plant species identified in the study area are reported in other regions of Lebanon [17, 18, 58], other Eastern Mediterranean [59, 60], and Middle East countries [61] to treat the same or different ailments. Such wide ethnomedicinal uses of these species for similar or different conditions in many countries are a well-founded indication of high therapeutic potential of these species. A comparison between the findings of current study and those of Hermon Mountain, a recently announced reserve, in the Southeast of the country shows a rich uniqueness in medicinal species and associated knowledge of SBR. Examples of unique uses are the decoction of flowers and fruits of *Crataegus azarolus* L. for diabetes as well as seeds and roots of *Sarcopoterium spinosum* (L.) Spach, fresh leaves of *Dittrichia viscosa* for wounds healing, decoction of aerial parts of *Daucus carota* for kidney stones, uterine and urinary infections, and whole plant of *Eryngium creticum* for snake /insect bites and skin diseases. On the other hand, the similarity observed can be explained by vegetation resemblance as well as the high cultural exchange between the local communities of SBR and Hermon Mountain.

The high utilization of leaves is in line with previous research in Lebanon [17] and also in other countries [62]. Leaves are favored parts in the preparation of herbal formulation because of their easy handling and sustainability. Leaves also contain diverse plant secondary metabolites. However, other plant parts such as whole plants and roots were also frequently cited. This practice can cause a decline in the plant populations and affect its natural regeneration, especially that the majority of cited plants were of herbaceous nature. On the other hand, removing leaves within reasonable limits does not interfere with the plant life compared to collecting other parts that may risk plant lives.

### Frequent diseases and cited medicinal plants

Analysis of FIC data (Table 4) shows that 24 plant species were reported to treat cardiovascular diseases being the main cause of death in Lebanon [63]. Among these species are *Crataegus azarolus* L., *Crataegus monogyna* Jacqu., *Juniperus excelsa* M.Bieb., *Paronychia echinulata* Chater, Papaver rhoeas L., and *Prunus prostrata* Labill. Also, 142 and 83 plant species were reported to treat gastrointestinal and respiratory diseases, respectively. This clearly reflects the importance of the cited species in controlling the spread of some CDs of these systems reported to show increases in prevalence in recent years [10].

### Mode of preparation, administration methods, and dosage of application

The frequent use of decoction might be due to the belief that heat is effective in the destruction of plant tissues leading to the release of many active compounds in water [64]. Compared with infusion, decoction is particularly very effective in extracting non-aromatic plants, and it, at the same time, preserves the herbal remedies for a longer period than fresh preparations. However, both decoction and infusion do not offer long shelf life for the preparations. Therefore, users would need to continuously harvest medicinal plants putting them under increased pressure of over harvesting.

Oral application as the main administration route is commonly used in many herbal remedies as reported in the literature [65]. It offers a large effective surface area for absorption of formulations' active components. This choice may be associated with the use of some solvents such as water, olive oil, almond oil, and honey. Such solvents and additives are commonly believed to serve as an efficient vehicle to transport remedies, enhance extraction of bioactive molecules, improve taste, and diminish adverse effects such as vomiting and diarrhea.

A lack of consistency regarding the dosage of formulations was apparent among informants. It was apparent that the traditional practitioners were more confident to provide more information about doses. However, informants reported that the use of plant species depends on several factors such as the nature of the plant, the user's age, health, and several other conditions. Other informants expressed high uncertainty. For example, few informants mentioned dosage and detailed formulation on the use of *Daucus carota* which is one of the most cited plants by informants for kidney stones and intestinal infections.

The general awareness of informants on the risk of some side effects and health complications to happen causing both short-term and long-term problems for patients is noteworthy. The literature provides many examples of cases about toxicity and safety implications of herbal medicines [66] attributed to many toxins. For example, *Tussilago farfara* L. contains toxic compounds among which pyrrolizidine alkaloids and *Prunus cocomilia* contains cyanogenic glycosides, especially amygdalin and prunasin [67]. Also*, Eupatorium cannabinum* contains hepatotoxic pyrrolizidine alkaloids which may block blood flow in the veins and cause liver damage, cancer, and breath defects [68]. Even when applied to broken skin, dangerous chemicals can be absorbed and can lead to dangerous body wide toxicity. Obviously, external use is relatively less risk as compared to internal/oral applications. Under any condition, there is a high chance of the patient to be a victim of the side effects of a medicinal plant. Importantly, the findings of this study do not entail any recommendations for the use of any of the cited plants or remedies. On the contrary, the safety concerns expressed by informants clearly conclude that there is a necessity for proper handling of the use medicinal plants and licensing of the practice of herbal medicine in Lebanon. This is especially important with the increasing tendency of communities to turn to herbal medicine under current economic crises and diminished healthcare services in the country. Previous research reports adverse effects that may be life-threatening in some cases, consequentially related to taking of herbal remedies [69]. Therefore, studies on the safety and efficacy involving analysis of the effectiveness of herbs are a necessity to ascertain efficacy and determine whether a cause-and-effect relation between therapeutic and health outcomes and treatment exists [70]. It is certain that herbal remedies and not only new synthetic drugs are required to meet international standards on quality, safety and efficacy [71]. Thus, there is a need to develop standardized traditional treatment guidelines for medicinal plants and to always keep in mind that natural products are not always necessarily safe. In this respect, the WHO regulations on medicinal plants may present a good baseline for such regulations [72].

Moreover, findings also indicate that several of the cited plants serve for various purposes such as food, bee forage, and pesticides increasing their potential for community livelihood.

### Quantitative indices

Species with the highest FC and RFC values and those of high UV specify the usefulness of these species and their potential as priority species that merit future comprehensive phytochemical and pharmacological studies [73]. Some of these species such as *Dittrichia viscosa, Daucus carota*, *Capparis spinosa,* and *Micromeria myrtifolia* scored high FC and RFC values in previous reports from different parts of the world [74, 75]. This confirms the high therapeutic potential of such species as an important alternative source to provide people with formulations to prevent disease, maintain health or cure ailments. On the other hand, some of the herein cited species are unique being narrow endemics to Lebanon and Syria or the region. Among these are *Pseudopodospermum libanoticum*, locally named as Dabeh Lebnan, *Arum palaestinum* (Loof), and *Berberis libanotica *(Berbaris loubnani) [76, 77].

The maximum FIC value reported for gastrointestinal disorders may reflect the widespread prevalence of these diseases in the area under study as well as the efficacy of cited plants in the treatment of such diseases. Similar results for gastrointestinal disorders were also reported by other studies in the country [17].

The plant species *Dittrichia viscosa* with FL value of 100% has been reported by other authors confirming the potential of this plant to treat a particular ailment [73]. Richness in several metabolites such as sesquiterpenes, flavonoids and caffeic acids [49] explains the wide range of usage of *D. viscosa* for several ailments under many categories. Such plant should be considered for further phytopharmacological investigation.

## Conclusion and recommendations

The results of this ethnobotanical survey in the SBR show that a high diversity of medicinal plants and a wealth of traditional knowledge continue to play a significant role in the traditional healthcare system and the treatment of a wide range of diseases in the region.

The high dependency on medicinal plants for primary healthcare call for preserving these resources and the associated traditional knowledge, while protecting medicinal plants as an integral part of the SBR management plan.

Most medicinal plant species used belong to Asteraceae, Lamiaceae, and Rosaceae, frequently used to manage serious diseases worldwide. There was a general consensus on the plant species used to manage diseases of the gastrointestinal system. This is a clear indication that such plants are effective and merit further studies for phytochemical screening and identification of bioactive constituents and their associated pharmacological activities.

Despite community awareness of toxic side effects, careful attention and the establishment of regulations that govern the use of medicinal plants should be developed. Moreover, a uniform database (a Middle Eastern Pharmacopeia) on the characteristics and chemical profiles of commonly used species, their applications, efficacy and toxicity is a key measure to protect users against negative impacts.

There is a need to develop training programs on the sustainable use of medicinal plants for the SBR local communities and incorporate regulations to safeguard the diversity of medicinal plants as an important source of ecosystem services. This approach is critical to balance demand for medicinal plants and promote social well-being while maintaining the vitality of SBR ecosystem services.

The importance of traditional knowledge of medicinal plants was highly perceived by informants owing to the substantial role of culture in inspiring the conservation of medicinal plants. Therefore, SBR managers and authorities are invited to incorporate traditional knowledge into the Reserve's biodiversity conservation plan and management strategy.

## Supplementary Information

**Additional file 1.** Table S1 : Quantitative and qualitative ethnobotanical data of wild medicinal plants in the Shouf Biosphere Reserve, Lebanon.

## Data Availability

All raw data, herbarium specimens, photographs, videos, scanned herbarium specimens, etc. are available.
